# Secular Decreasing Trend in Plasma Eicosapentaenoic and Docosahexaenoic Acids among Patients with Acute Coronary Syndrome from 2011 to 2019: A Single Center Descriptive Study

**DOI:** 10.3390/nu13010253

**Published:** 2021-01-17

**Authors:** Tomoaki Okada, Toru Miyoshi, Masayuki Doi, Kosuke Seiyama, Wataru Takagi, Masahiro Sogo, Kazumasa Nosaka, Masahiko Takahashi, Keisuke Okawa, Hiroshi Ito

**Affiliations:** 1 Department of Cardiology, Kagawa Prefectural Central Hospital, 1-2-1 Asahi-machi, Takamatsu City 760-8557, Japan; ju_10monomoto0622@yahoo.co.jp (T.O.); mdoimd@gmail.com (M.D.); k.seiyama9543@gmail.com (K.S.); t_wataru1206@yahoo.co.jp (W.T.); msogo0517@gmail.com (M.S.); kn_orch@yahoo.co.jp (K.N.); masahiko0707@infoseek.jp (M.T.); drop-in-bigriver@hotmail.co.jp (K.O.); 2 Department of Cardiovascular Medicine, Okayama University Graduate School of Medicine, Dentistry and Pharmaceutical Sciences, 2-5-1 Shikata-cho, Okayama City 700-8558, Japan; itomd@md.okayama-u.ac.jp

**Keywords:** atherosclerotic cardiovascular disease, polyunsaturated fatty acids, eicosapentaenoic acid, docosahexaenoic acid, arachidonic acid, descriptive study

## Abstract

Despite intensive lipid-lowering interventions, patients treated with statins develop atherosclerotic cardiovascular disease (ASCVD), and these patients have an increased risk of developing recurrent cardiovascular events during follow-up. Therefore, there is a need to focus on the residual risks in patients in statin therapy to further reduce ASCVD. The aim of this study was to retrospectively investigate the 10-year trend (2011–2019) regarding changes in polyunsaturated fatty acids (PUFAs) in patients with acute coronary syndrome (ACS) in a single center. We included 686 men and 203 women with ACS admitted to Kagawa Prefectural Central Hospital. Plasma PUFAs, including eicosapentaenoic acid (EPA), docosahexaenoic acid (DHA), arachidonic acid (AA), and dihomo-γ-linolenic acid (DGLA), were measured at admission for suspected ACS. A secular decreasing trend in the levels of EPA and DHA and the EPA/AA ratio, but not of AA and DGLA, was observed. The analyses based on age (>70 or <70 years) and sex showed that the decreasing trend in the levels of EPA and DHA did not depend on age and remained significant only in men. Further studies are needed to obtain robust evidence to justify that the administration of n-3 PUFA contributes to the secondary prevention of ACS.

## 1. Introduction

Atherosclerotic cardiovascular disease (ASCVD) is the leading cause of mortality, accounting for 30% of all global deaths [1]. There are several methods to prevent ASCVD, including smoking cessation, increased physical activity, and weight loss [2]. The recent guidelines for the prevention of ASCVD recommend lipid-lowering agents, particularly statins, as the essence of primary and secondary prevention of ASCVD [3,4,5]. However, despite intensive lipid-lowering interventions, patients treated with statins develop ASCVD, and these patients have an increased recurrent risk of cardiovascular events [6,7,8]. Therefore, the residual risks in patients on statin therapy need to be the focus to further reduce ASCVD. An observational study showed that lower n-3 polyunsaturated fatty acids (PUFAs), especially eicosapentaenoic acid (EPA) and docosahexaenoic acid (DHA), were associated with the incidence of ASCVD [9]. Other studies have demonstrated that a low ratio of EPA to arachidonic acid (AA) is associated with a greater risk of cardiovascular disease [10,11,12]. Therefore, active screening of PUFAs is beneficial in identifying patients at high risk for ASCVD. A survey of a community-dwelling middle-aged and elderly Japanese population over 13 years from 1997 to 2012 showed that EPA and DHA concentrations increased in those aged over 60 years [13]. However, there is no data on the secular trend of PUFAs in patients with ASCVD. To establish appropriate intervention for secondary prevention, more recent data, especially in patients with ASCVD, is needed.

Therefore, we investigated the 10-year trend regarding changes in PUFAs in patients with acute coronary syndrome (ACS) based on data from a single center.

## 2. Materials and Methods

This was a single-center, retrospective, observational study. We enrolled 889 patients who were treated for ACS at the Kagawa Prefectural Central Hospital between January 2011 and December 2019. All these patients were evaluated for plasma EPA, DHA, AA, and dihomo-γ-linolenic acid (DGLA) on the day of admission for suspected ACS. ACS was diagnosed according to the American College of Cardiology/American Heart Association 2007 guideline; recent-onset chest pain, associated with ST segment and/or negative T wave electrocardiogram (ECG) changes and/or positive cardiac enzymes (creatine kinase or troponin T) [14]. Patients were excluded if they were administered pure EPA formulations.

This study was approved by the ethics committee of Kagawa Prefectural Central Hospital. The requirement for informed consent was waived because of the low-risk nature of the study and the inability to obtain consent directly from all the study subjects. Instead, we announced this study protocol extensively at Kagawa Prefectural Central Hospital and on the hospital website (http://www.chp-kagawa.jp/) and provided patients with the opportunity to withdraw from the study. The study was conducted in accordance with the principles of the Declaration of Helsinki.

### 2.1. Blood Sampling

Blood samples were obtained in the emergency room, and plasma levels of EPA, DHA, AA, and DGLA were measured at an external laboratory (SRL Inc., Tokyo, Japan). Routine laboratory tests, including total cholesterol, fasting triglycerides, low-density lipoprotein cholesterol (LDL-C), high-density lipoprotein cholesterol (HDL-C), hemoglobin A1c, and serum creatinine, were performed using an automated analyzer at Kagawa Prefectural Central Hospital. LDL concentration was assayed directly.

### 2.2. Assessment of Additional Risk Factors

Hypertension was confirmed according to the Japanese Society of Hypertension Guidelines for the Management of Hypertension 2014 [15]. Diabetes mellitus was defined as having a previous diagnosis of diabetes mellitus in the medical records, having a hemoglobin A1c (national glycohemoglobin standardization program calculation) level ≥ 6.5%, or receiving treatment with oral antidiabetic agents or insulin. Dyslipidemia was defined according to the Japan Atherosclerosis Society Guidelines for Prevention of Atherosclerotic Cardiovascular Diseases 2017 [16]. Smoking status was defined as currently smoking.

### 2.3. Statistical Analysis

Continuous variables are presented as mean ± standard deviation or mean ± 95% confidence interval (CI). Categorical variables are presented as frequency and proportion (%). The observation period was divided into three terms: the first term (2011–2013), second term (2014–2016), and third term (2017–2019). Differences among groups were evaluated using the chi-square test for categorical variables, and differences in continuous variables were compared by analysis of variance. Analysis of covariance allowed the comparison of PUFAs among groups while taking into account age and sex. For multiple comparisons, the Bonferroni post-hoc test was applied. A 2-tailed *p*-value of <0.05 was considered statistically significant. All statistical analyses were performed using SPSS 27.0 for Windows (IBM, Armonk, NY, USA).

## 3. Results

Analysis of the patient characteristics and lipid profile of the study population according to the terms as shown in Table 1, patients in the third term were significantly older than those in the second term. The prevalence of dyslipidemia in the third term was significantly lower than that in the second term. There was a higher prevalence of patients with current smoking habit and statin use in the second term than in the first and third terms, respectively. The level of hemoglobin A1c in the second term was significantly lower than that in the first term. The level of triglyceride in the third term was significantly lower than that in the first term, whereas the levels of LDL-C and HDL-C did not differ among the three terms.

On analyzing the secular trend of EPA, AA, DGLA, AA, and EPA/AA levels in all patients, and according to age (>70 or < 70years) and sex (Table 2), the levels of EPA and DHA in the third term were significantly lower than those in the first and second terms, respectively. The AA level in the third term was significantly higher than that in the first term. The DGLA level in the second term was significantly higher than that in the first and third terms. Regarding the influence of age on the trend, the levels of EPA and DHA in the third term remained significantly lower than those in the first term in both patients aged <70 years and >70 years. Regarding the influence of sex on the trend, the levels of EPA and DHA in the third term remained significantly lower than those in the first term in men, but not in women. The levels of AA and DGLA did not differ among the three terms regardless of age and sex. The EPA/AA ratio in the third term was significantly lower than that in the first term regardless of age, and this difference remained significant in men, but not in women.

Next, the secular trends of EPA, AA, DGLA, and EPA/AA levels in patients with and without diabetes were analyzed separately (Table 3 and Table 4). The trend in patients with diabetes was similar to that in all patients. However, in patients without diabetes, the levels of EPA in the third term were significantly lower than those in the second term in women >70 years; the levels of DHA in the third term were significantly lower than those in the second term regardless age and sex; and the EPA/AA ratio in the third term was significantly lower than that in the first and the second terms in women >70 years.

**Figure 1:**
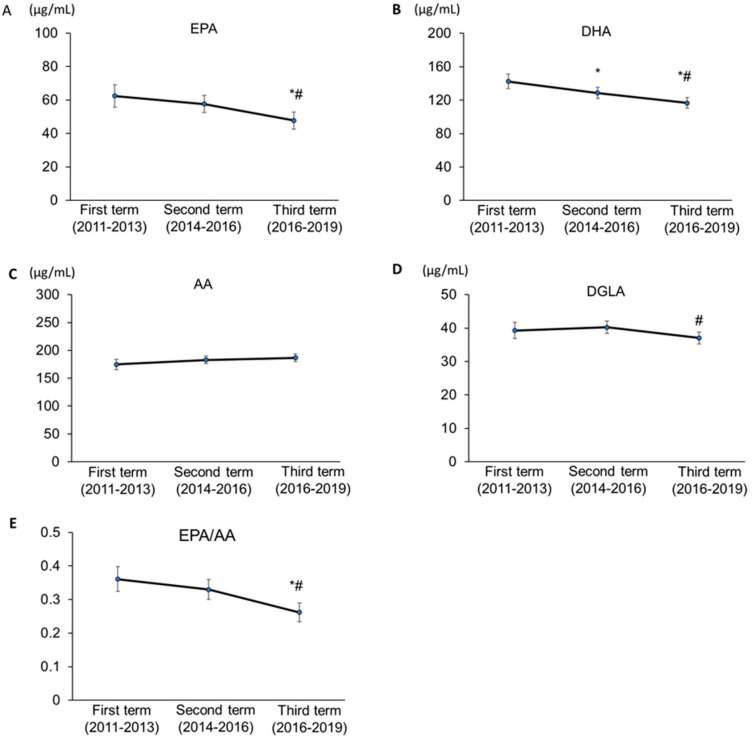
Secular trend in age- and sex- adjusted polyunsaturated fatty acids and EPA/AA. (**A**) EPA, (**B**) DHA, (**C**) AA, (**D**) DGLA, and (**E**) EPA/AA Data are presented as mean ± 95% confidence interval. EPA, eicosapentaenoic acid; DHA, docosahexaenoic acid; DGLA, dihomo-γ-linolenic acid; AA, arachidonic acid. * *p* < 0.05 versus first term and ^#^ *p* < 0.05 versus second term. Analysis of covariance allowed the comparison of PUFAs among groups while taking into account age and sex. For multiple comparisons, the Bonferroni post-hoc test was applied.

Based on Figure 1, which shows the secular trend in the age- and sex-adjusted EPA, AA, DGLA, and EPA/AA levels, the adjusted levels of PUFAs [mean (95% CI)] in the first, second, and third terms were 62.4 (55.5–69.2), 57.6 (52.4–62.8), and 47.7 (42.6–52.8) of EPA (μg/mL, *p* = 0.001); 142.4 (133.9–150.6), 128.7 (122.3–13.5.1), and 116.6 (110.3–122.9) of DHA (μg/mL, *p* < 0.001); 174.9 (165.7–14.0), 182.7 (175.8–189.6), 186.8 (180.1–193.6) of AA (*p* = 0.102); 39.2 (36.8–41.7), 40.2 (38.4–42.0), and 37.0 (35.2–38.8) of DGLA (μg/mL, *p* = 0.045), 0.63 (0.32–0.40), 0.33 (0.30–0.36), and 0.26 (0.23–0.29) of EPA/AA (*p* < 0.001), respectively.

## 4. Discussion

This study demonstrated a decreasing trend in the levels of EPA, DHA, and EPA/AA, but not of AA and DGLA. Analyses based on age (<70 and >70 years) and sex showed that this decreasing trend in the levels of EPA and DHA did not depend on age and was significant only in men. To our knowledge, this is the first study to describe changes in PUFAs over a decade in patients with ACS.

A previous study from 1997 to 2012 in a contemporary healthy Japanese population living in an urban area showed a secular increasing trend in the serum levels of EPA and DHA in the population aged over 60 years [17]. The present study evaluated subsequent changes in PUFAs from 2011 to 2019 in patients with ACS and demonstrated a decreasing trend in plasma EPA and DHA. After 2010, a substantial reduction in the fish intake in the Japanese elderly population was reported [18], which could be an explanation of this decreasing trend of n-3 PUFAs in the present study, as fish intake is closely correlated with circulating n-3 PUFAs level [19]. Another explanation is the difference in participant characteristics between the general population and patients with ACS. A previous study in patients with ACS showed that the mean levels of EPA and DHA during 2004 and 2011 were 73.4 μg/mL and 146.9 μg/mL, respectively, which were slightly higher than those in the first term (2011–2013) in our study (63.0 μg/mL and 138.1 μg/mL, respectively). Furthermore, the level of AA during 2004 and 2011 in patients with ACS was reported to be 159.9 μg/mL, which was moderately lower than that in the first term in the present study of 169.6 μg/mL. Thus, in patients with ACS, a decreasing trend in the levels of EPA, DHA, and EPA/AA may have continued before 2011. However, further larger studies are needed to confirm this decreasing trend in patients with ACS.

The mechanisms underlying the favorable effects of n-3 PUFAs remain partly unknown [20], whereas previous experimental studies showed that n-3 PUFAs have multiple actions towards prevention of ASCVD, including anti-inflammatory effect [21], inhibition of platelet aggregation [22], and improvement of endothelial function [23]. Several studies using intra-coronary imaging showed that circulating EPA levels were associated with lipid plaque volume, [24] and fibrous-cap thickness of the plaque [25]. A randomized control study evaluating coronary plaque change by multidetector computed tomography showed that treatment with n-3 PUFA formulation for 18 months significantly reduced the accumulation of low-attenuation plaque, which is a component of vulnerable plaque, compared to placebo [26]. Furthermore, a recent study showed that the proportion of EPA in serum phosphatidylcholine at the time of ACS was associated with further clinical adverse events [27]. Thus, clinical trials and basic experiments suggest an important association between n-3 PUFAs and the development of ASCVD.

The Japanese Registry of All Cardiac and Vascular Disease showed that the number of patients with ACS is slightly increasing in Japan (http://www.j-circ.or.jp/jittai_chosa/). Furthermore, despite significant improvement in cardiovascular events by intensive statin treatment, up to 40% of statin-treated patients continue to experience recurrent cardiovascular events [8]. Thus, the control of risk factors beyond LDL-C and residual risk is an emerging problem for preventing ASCVD. In this context, n-3 PUFA has been evaluated as a residual cardiovascular risk factor. Our study demonstrated the secular decreasing trend in the levels of EPA, DHA, and EPA/AA without significant changes in LDL-C and HDL-C. Although our study did not evaluate the association between the secular decreasing trend and the incidence of ASCVD, it highlights the importance of n-3 PUFAs in the development of ACS.

Previous studies have shown that triglyceride-rich lipoproteins, the main carriers of triglycerides, are associated with foam cell formation via macrophage uptake directly at the arterial wall, which results in the development of atherosclerosis [28]. In addition, recent genetic studies have provided robust evidence of the role of triglycerides in the causal pathway for ASCVD [29]. Thus, supplemental n-3 PUFA lower triglyceride can be used as a treatment in patients with hypertriglyceridemia. However, the clinical benefit of the treatment of hypertriglyceridemia for the prevention of ASCVD has not been established. Future clinical trials are needed to determine whether triglyceride-lowering therapies reduce the risk of ASCVD in patients with hypertriglyceridemia. In addition, lipoprotein(a), which is composed of an LDL-like particle and characteristic glycoprotein apolipoprotein(a) connected by a disulfide bond, has been recognized as a residual risk of ASCVD [30]. Thus, the control of residual risks is an emerging issue to prevent ASCVD after the achievement of substantial reduction of LDL-C.

Although a few clinical studies have shown the effectiveness of n-3 PUFAs in high-risk patients, the benefit of n-3 PUFAs in the prevention of ASCVD remains controversial [31,32,33,34,35,36,37,38,39,40,41,42]. For primary prevention, a study of cardiovascular events in diabetes (ASCEND) [43] and a long-term outcomes study to assess STatin Residual Risk Reduction With EpaNova in HiGh Cardiovascular Risk PatienTs With Hypertriglyceridemia (STRENGTH) [44] failed to show a significant reduction in ASCVD by n-3 PUFA formulation in high risk patients. The Vitamin D and Omega-3 Trial (VITAL) showed that supplemental n-3 PUFAs did not significantly reduce the primary cardiovascular end point of major cardiovascular events (composite of myocardial infarction, stroke, and cardiovascular mortality), but were associated with significant reductions in total myocardial infarction, percutaneous coronary intervention, and fatal myocardial infarction [45]. On the other hand, for secondary prevention, Reduction of Cardiovascular Events With EPA—Intervention Trial (REDUCE-IT) [46] showed a significant reduction in ASCVD. There were several differences among the studies. One issue is the formulation of n-3 PUFAs. The ASCEND, the VITAL, and the STRENGTH tested mixed formulations of EPA and DHA, whereas the REDUCE-IT evaluated pure EPA formulation. Another issue is the dose of n-3 PUFA. Participants in the ASCEND and the VITAL received 1 g/day of n-3 PUFA, whereas participants in the STRENGTH and the REDUCE-IT received 4 g/day. A high dose of n-3 PUFA formulation may be preferable because circulating EPA levels were shown to be tightly linked with ASCVD outcomes in the REDUCE-IT trial [46]. Furthermore, it is possible that the specific formulation of n-3 PUFAs makes a difference because the biological roles of EPA and DHA in tissue are different [18]. Further large-scale, carefully planned, and controlled clinical studies are needed to provide solid evidence that n-3 PUFAs can prevent ASCVD.

There are several limitations in this study. This study was retrospectively performed at a single center located in a costal provincial city. Therefore, the study results cannot be applied directly to patients living in urban areas. Second, this study did not aim to analyze the causal relationship between the decrease in the levels of EPA and DHA, EPA/AA, and the incidence of ACS. Further investigation is needed to achieve robust evidence on the role of n-3 PUFAs in preventing ASCVD.

## 5. Conclusions

This study demonstrated a decreasing trend in the levels of EPA and DHA, and EPA/AA in men from 2011 to 2019 without significant changes in the levels of LDL-C and HDL-C. This decreasing trend in the levels of EPA and DHA did not depend on age and was significant only in men. Considering the results of recent large-scale trials, administration of a sufficient dose of n-3 PUFAs may contribute to the secondary prevention of ACS, but further studies are needed to obtain robust evidence.

## Figures and Tables

**Table 1:** Patient characteristics in the first, second and third terms.

	First Term (2011–2013), *n* = 238	Second Term (2014–2016), *n* = 285	Third Term (2017–2019), *n* = 366	*p* Value
Age, years	70.3 ± 11.4	68.5 ± 12.1	71.7 ± 12.7 ^#^	0.003
Male/female	181 (76)/57 (24)	227 (80)/58 (20)	278 (76)/88 (24)	0.480
Body mass index, kg/m^2^	23.7 ± 3.8	23.9 ± 3.4	23.8 ± 3.8	0.765
Hypertension	165 (69)	211 (74)	264 (72)	0.489
Diabetes mellitus	92 (39)	95 (33)	140 (38)	0.340
Dyslipidemia	159 (67)	199 (70)	219 (60) ^#^	0.023
Current smoker	69 (29)	124 (43.8) *	107 (29) ^#^	<0.001
AMI/UAP	160 (67)/78 (33)	194 (68)/91 (32)	245 (67)/121 (33)	0.097
History of CAD	37 (16)	32 (11)	47 (13)	0.34
Statin	39 (16)	74 (26) *	67 (18) ^#^	0.012
Ezetimibe	N/A	5 (2)	7 (2)	0.710
ACEI/ARB	65 (28)	108 (37)	106 (28)	0.770
β-blocker	22 (9)	28 (10)	31 (8)	0.995
Serum creatinine, mg/dL	1.00 ± 0.91	0.98 ± 0.88	1.18 ± 1.49	0.057
Hemoglobin A1c, %	6.4 ± 1.3	6.0 ± 0.9 *	6.2 ± 1.1	0.005
Total cholesterol, mg/dL	181 ± 39	185 ± 44	180 ± 40	0.223
LDL-C, mg/dL	114 ± 33	116 ± 36	113 ± 34	0.692
Triglyceride, mg/dL	110 ± 131	114 ± 99	94 ± 77 ^#^	0.037
HDL-C, mg/dL	42 ± 10	44 ± 12	44 ± 12	0.257
EPA, μg/mL	63.0 ± 42.2	60.2 ± 38.3	51.6 ± 33.2 *^#^	<0.001
DHA, μg/mL	138.1 ± 51.7	130.2 ± 51.0	116.0 ± 43.8 *^#^	<0.001
AA, μg/mL	169.6 ± 45.6	178.3 ± 53.2	180.8 ± 55.7 *	0.032
DGLA, μg/mL	36.0 ± 14.7	39.3 ± 15.2 *	36.0 ± 14.8 ^#^	0.010
EPA/AA	0.37 ± 0.22	0.35 ± 0.22	0.29 ± 0.18 *^#^	<0.001

Categorical variables are presented as number of patients (%). Continuous variables are presented as mean ± standard deviation. AMI, acute myocardial infarction; UAP, unstable angina pectoris; CAD, coronary artery disease; N/A, not available; ACEI/ARB, angiotensin-converting-enzyme inhibitor/angiotensin II Receptor Blocker; HDL-C, high-density lipoprotein cholesterol; LDL-C, low-density lipoprotein cholesterol; EPA, eicosapentaenoic acid; DHA, docosahexaenoic acid; DGLA, dihomo-γ-linolenic acid; AA, arachidonic acid. * *p* < 0.05 versus first term and ^#^ *p* < 0.05 versus second term. For multiple comparisons, the Bonferroni post-hoc test was applied.

**Table 2:** Secular trend in polyunsaturated fatty acids in all patients.

	First Term (2011–2013)	Second Term (2014–2016)	Third Term (2017–2019)	*p* Value
EPA, μg/mL				
All patients	63.0 ± 42.2	60.2 ± 38.3	51.6 ± 33.2 *^#^	<0.001
Age ≤ 70 years	62.8 ± 40.1	57.9 ± 37.4	49.5 ± 31.2 *	0.010
Age > 70 years	65.9 ± 50.4	62.6 ± 36.6	53.4 ± 34.9 *	0.018
Men	64.5 ± 43.9	58.8 ± 36.7	51.7 ± 31.7 *	0.002
Women	63.8 ± 50.3	64.8 ± 38.3	51.3 ± 37.8	0.080
DHA, μg/mL				
All patients	138.1 ± 51.7	130.2 ± 51.0	116.0 ± 43.8 *^#^	<0.001
Age ≤ 70 years	142.9 ± 62.5	128.6 ± 49.0	110.9 ± 41.9 *	<0.001
Age > 70 years	139.0 ± 48.1	131.6 ± 48.4	120.2 ± 45.1 *	0.004
Men	140.5 ± 59.1	126.3 ± 46.0 *	111.7 ± 38.9 *^,#^	<0.001
Women	142.2 ± 44.4	143.9 ± 56.0	129.4 ± 54.7	0.183
AA, μg/mL				
All patients	169.6 ± 45.6	178.3 ± 53.2	180.8 ± 55.7 *	0.032
Age ≤ 70 years	179.4 ± 53.1	185.7 ± 53.2	192.0 ± 61.7	0.225
Age > 70 years	161.9 ± 39.4	164.7 ± 46.4	171.5 ± 48.4	0.186
Men	169.7 ± 48.1	171.3 ± 50.5	179.4 ± 53.3	0.093
Women	174.1 ± 45.8	194.8 ± 50.3	185.5 ± 63.1	0.150
DGLA, μg/mL				
All patients	36.0 ± 14.7	39.3 ± 15.2 *	36.0 ± 14.8 ^#^	0.010
Age ≤ 70 years	40.1 ± 17.2	42.8 ± 16.0	39.6 ± 14.3	0.124
Age > 70 years	32.4 ±12.7	33.5 ± 11.1	33.1 ± 14.6	0.813
Men	36.6 ± 15.6	38.2 ± 15.0	36.2 ± 15.6	0.298
Women	35.2 ± 15.5	40.1 ± 13.4	35.6 ± 12.2	0.066
EPA/AA				
All patients	0.37 ± 0.22	0.35 ± 0.22	0.29 ± 0.18 *^,#^	<0.001
Age ≤ 70 years	0.35 ± 0.19	0.32 ± 0.20	0.27 ± 0.17 *	0.002
Age > 70 years	0.40 ± 0.28	0.439 ± 0.23	0.31 ± 0.19 *	0.001
Men	0.38 ± 0.24	0.36 ± 0.21	0.30 ± 0.21 *	<0.001
Women	0.35 ± 0.22	0.35 ± 0.23	0.27 ± 0.17	0.035

Continuous variables are presented as mean ± standard deviation. EPA, eicosapentaenoic acid; DHA, docosahexaenoic acid; DGLA, dihomo-γ-linolenic acid; AA, arachidonic acid. * *p* < 0.05 versus first term and ^#^ *p* < 0.05 versus second term. For multiple comparisons, the Bonferroni post-hoc test was applied.

**Table 3:** Secular trend in polyunsaturated fatty acids in patients with diabetes.

	First Term (2011–2013), *n* = 92	Second Term (2014–2016), *n* = 95	Third Term (2017–2019), *n* = 139	*p* Value
EPA, μg/mL				
Age ≤ 70 years	71.2 ± 44.3	58.1 ± 32.2	46.3 ± 28.2 *^,#^	0.001
Age > 70 years	71.4 ± 50.5	62.5 ± 40.8	54.3 ± 41.4 *	0.125
Men	71.6 ± 49.3	59.9 ± 37.8	48.2 ± 28.5 *^,#^	<0.001
Women	70.2 ± 37.1	61.4 ± 31.8	60.0 ± 56.1	0.732
DHA, μg/mL				
Age ≤ 70 years	145.4 ± 67.4	127.7 ± 46.8	106.2 ± 43.7 *^,#^	0.001
Age > 70 years	146.3 ± 51.1	131.4 ± 58.3	120.6 ± 54.7 *	0.055
Men	144.6 ± 65.0	125.9 ± 47.8 *	106.6 ± 37.1 *^,#^	<0.001
Women	150.4 ± 38.8	142.9 ± 66.4	142.1 ± 77.6	0.903
AA, μg/mL				
Age ≤ 70 years	183.0 ± 58.8	239.4 ± 331.6	194.8 ± 70.9	0.297
Age > 70 years	162.6 ± 36.6	173.0 ± 38.6	170.4 ± 42.5	0.455
Men	173.0 ± 52.6	210.2 ± 272.1	178.3 ± 50.9	0.266
Women	177.1 ± 45.1	199.6 ± 64.4	191.2 ± 78.1	0.569
DGLA, μg/mL				
Age ≤ 70 years	42.6 ± 19.2	44.5 ± 16.4	39.9 ± 14.4	0.348
Age > 70 years	32.5 ± 12.2	34.7 ± 12.8	31.2 ± 11.0	0.280
Men	38.2 ± 17.7	39.8 ± 15.1	34.7 ± 13.1 ^#^	0.063
Women	37.8 ± 15.3	40.1 ± 17.5	36.2 ± 13.9	0.694
EPA/AA				
Age ≤ 70 years	0.38 ± 0.20	0.31 ± 0.19	0.25 ± 0.14 *^,#^	<0.001
Age > 70 years	0.45 ± 0.33	0.36 ± 0.24	0.31 ± 0.19 *	0.016
Men	0.42 ± 0.27	0.34 ± 0.23	0.28 ± 0.16 *^,#^	<0.001
Women	0.40 ± 0.23	0.31 ± 0.14	0.30 ± 0.23	0.220

Continuous variables are presented as mean ± standard deviation. EPA, eicosapentaenoic acid; DHA, docosahexaenoic acid; DGLA, dihomo-γ-linolenic acid; AA, arachidonic acid. * *p* < 0.05 versus first term and ^#^ *p* < 0.05 versus second term. For multiple comparisons, the Bonferroni post-hoc test was applied.

**Table 4:** Secular trend in polyunsaturated fatty acids in patients without diabetes.

	First Term (2011–2013), *n* = 146	Second Term (2014–2016), *n* = 190	Third Term (2017–2019), *n* = 227	*p* Value
EPA, μg/mL				
Age ≤ 70 years	54.9 ± 29.5	57.7 ± 42.6	51.3 ± 32.8	0.434
Age > 70 years	60.9 ± 45.4	63.2 ± 35.2	52.8 ± 30.0 ^#^	0.096
Men	56.8 ± 32.1	58.6 ± 38.6	53.9 ± 33.6	0.491
Women	60.7 ± 52.0	66.7 ± 42.2	46.8 ± 22.7 ^#^	0.036
DHA, μg/mL				
Age ≤ 70 years	134.7 ± 47.5	129.0 ± 54.2	113.7 ± 40.8 *^,#^	0.009
Age > 70 years	131.7 ± 42.3	132.3 ± 46.0	119.9 ± 37.8 *^,#^	0.057
Men	131.1 ± 45.1	127.1 ± 48.4	115.0 ± 39.9 *^,#^	0.006
Women	139.1 ± 44.3	144.3 ± 56.8	122.9 ± 37.1 ^#^	0.057
AA, μg/mL				
Age ≤ 70 years	175.0 ± 41.2	185.0 ± 53.1	190.3 ± 56.1 *	0.146
Age > 70 years	158.5 ± 41.0	163.7 ± 51.3	172.2 ± 52.0	0.151
Men	164.0 ± 40.1	169.8 ± 54.1	180.1 ± 54.8 *	0.031
Women	174.9 ± 45.9	197.1 ± 43.8 *	182.5 ± 54.3	0.137
DGLA, μg/mL				
Age ≤ 70 years	37.6 ± 13.1	43.4 ± 16.7 *	39.4 ± 14.3	0.027
Age > 70 years	31.6 ± 11.9	33.7 ± 10.7	34.3 ± 16.5	0.420
Men	34.7 ± 12.4	38.4 ± 15.7 *	37.2 ± 16.9	0.177
Women	34.4 ± 14.1	41.4 ± 11.8 *	35.3 ± 11.4 ^#^	0.024
EPA/AA				
Age ≤ 70 years	0.31 ±0.16	0.32 ± 0.22	0.28 ± 0.18	0.312
Age > 70 years	0.37 ± 0.22	0.40 ± 0.22	0.31± 0.18 *^,#^	0.006
Men	0.35 ± 0.18	0.35 ± 0.22	0.31 ± 0.20	0.101
Women	0.33 ± 0.22	0.36 ± 0.26	0.26 ± 0.12 ^#^	0.038

Continuous variables are presented as mean ± standard deviation. EPA, eicosapentaenoic acid; DHA, docosahexaenoic acid; DGLA, dihomo-γ-linolenic acid; AA, arachidonic acid. * *p* < 0.05 versus first term and ^#^ *p* < 0.05 versus second term. For multiple comparisons, the Bonferroni post-hoc test was applied.

## Data Availability

The data presented in this study are available on request from the corresponding author.
